# Riluzole Increases the Amount of Latent HSF1 for an Amplified Heat Shock Response and Cytoprotection

**DOI:** 10.1371/journal.pone.0002864

**Published:** 2008-08-06

**Authors:** Jingxian Yang, Kristen Bridges, Kuang Yu Chen, Alice Y.-C. Liu

**Affiliations:** 1 Department of Cell Biology and Neuroscience, Rutgers State University of New Jersey, Piscataway, New Jersey, United States of America; 2 Department of Chemistry and Chemical Biology, Rutgers State University of New Jersey, Piscataway, New Jersey, United States of America; Istituto Dermopatico dell'Immacolata, Italy

## Abstract

**Background:**

Induction of the heat shock response (HSR) and increased expression of the heat shock proteins (HSPs) provide mechanisms to ensure proper protein folding, trafficking, and disposition. The importance of HSPs is underscored by the understanding that protein mis-folding and aggregation contribute centrally to the pathogenesis of neurodegenerative diseases.

**Methodology/Principal Findings:**

We used a cell-based hsp70-luciferease reporter gene assay system to identify agents that modulate the HSR and show here that clinically relevant concentrations of the FDA-approved ALS drug riluzole significantly increased the heat shock induction of hsp70-luciferse reporter gene. Immuno-Western and -cytochemical analysis of HSF1 show that riluzole increased the amount of cytosolic HSF1 to afford a greater activation of HSF1 upon heat shock. The increased HSF1 contributed centrally to the cytoprotective activity of riluzole as hsf1 gene knockout negated the synergistic activity of riluzole and conditioning heat shock to confer cell survival under oxidative stress. Evidence of a post-transcriptional mechanism for the increase in HSF1 include: quantitation of mRNA^hsf1^ by RT-PCR showed no effect of either heat shock or riluzole treatment; riluzole also increased the expression of HSF1 from a CMV-promoter; analysis of the turnover of HSF1 by pulse chase and immunoprecipitation show that riluzole slowed the decay of [^35^S]labeled-HSF1. The effect of riluzole on HSF1 was qualitatively different from that of MG132 and chloroquine, inhibitors of the proteasome and lysosome, respectively, and appeared to involve the chaperone-mediated autophagy pathway as RNAi-mediated knockdown of CMA negated its effect.

**Conclusion/Significance:**

We show that riluzole increased the amount of HSF1 to amplify the HSR for cytoprotection. Our study provides novel insight into the mechanism that regulates HSF1 turnover, and identifies the degradation of HSF1 as a target for therapeutics intervention.

## Introduction

A common feature of many neurodegenerative diseases is the misfolding-due to genetic as well as epigenetic factors-of specific proteins, aggregation and formation of protein fibrillary structures termed amyloid inside and outside of brain cells [1]; terms such as “protein mis-folding diseases” and “proteinoapthies” have been coined to describe such disorders . There is also good evidence from *in vivo* studies in fruit fly and mouse models of the importance and relevance of heat shock protein chaperones (HSP) in preventing/mitigating such pathological consequences of protein mis-folding. In a *Drosophila* model, over-expression of human Hsp70 completely suppressed the external eye defects mediated by the expression of expanded polyQ protein, and partially restored retinal structure [2]. Conversely, expression of the expanded polyQ protein in a *Drosophila* line bearing a dominant-negative Hsp70 augmented the severity and kinetics of neurodegeneration, suggesting that under normal conditions the endogenous Hsp70 protein may partially mitigate the toxic effects of the expanded polyQ protein [2]. These considerations suggest that agents that upregulate the HSR and HSP chaperones may hold promise in therapeutics development for the prevention, management, and treatment of neurodegeneration [3], [4], [5], [6].

Riluzole (brand name Rilutek®, Sanofi-Aventis Inc.) is the first and thus far only FDA approved drug for the treatment of ALS (Amyotrophic lateral sclerosis; aka: Lou Gehrig's disease). Riluzole has a modest effect on the progression of ALS, it's mechanism of action is not well understood and may involve inhibition of glutamate release and excitotoxicity (http://products.sanofi-aventis.us/rilutek/rilutek.html) [7]. Importantly, the protective effect of riluzole is not limited to diseased motor neurons in ALS: riluzole confers neuroprotection in spinal cord and cortical injury/ischemia [8], [9], [10], [11], [12], retards *huntingtin* aggregate formation in a cell free system and hippocampi organ culture [13], slows the progression of multiple sclerosis in human [14], and retards neuromuscular dysfunction in wobbler mouse motor neuron disease [15].

We are interested in harnessing the cytoprotective function of the HSR and HSP chaperones. We developed a cell based hsp70-luciferase reporter gene assay to identify agents that can up-regulated the HSR. In particular, we are interested in candidates that are not proteotoxic and would not by themselves trigger the full HSR but nonetheless would enhance the effect of HSR elicitor. We show here that the FDA approved drug riluzole significantly amplified the effects of heat shock in induction of the hsp70-luciferse reporter gene expression. Analysis of the effects of riluzole on HSF1, the transcription factor that mediates the HSR, show that riluzole increased the amount of latent HSF1 monomer by blunting its turnover. The increased HSF1 reserve allowed for a more robust HSR to confer protection for survival under stress.

## Results

### Effects of riluzole on hsp70-reporter gene expression

**Figure 1:**
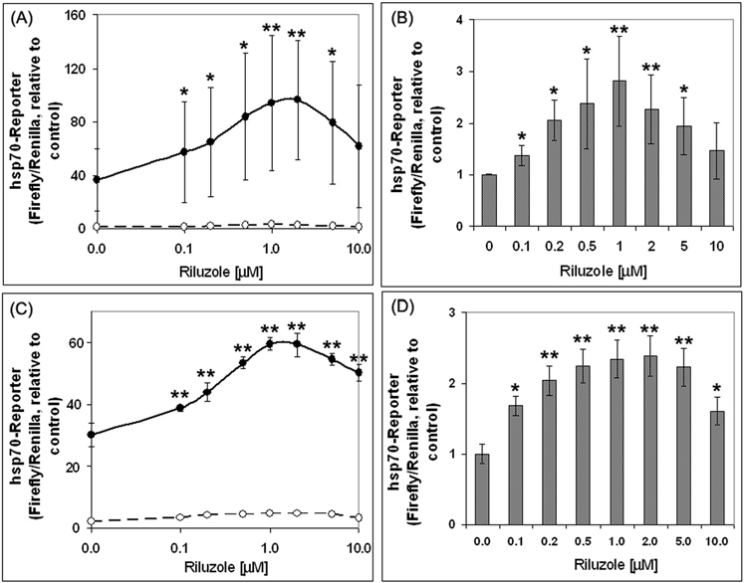
Dose-response effect of riluzole on the basal and heat shock induced hsp70-luciferase reporter gene expression. HeLa cells were transfected with the hsp 70-firefly luciferase reporter DNA and the internal control Renilla luciferase DNA according to methods described in the text. 6 hr after DNA transfection, cells were plated into 96 Stripwell® plates (Corning/Costar 9102). Riluzole was added to individual wells to final concentrations as indicated and incubated at 37°C for 16 hr. The condition used for heat shock was 2 hr at 42°C followed by recovery at 37°C for 4 hr. Controls were incubated at 37°C for an equivalent time. Luciferase activity was assayed using the Dual-Glo luciferase assay system from Promega (E2920) as described. Result on hsp70-reporter is presented as a ratio of hsp70-firefly luciferase over the internal control Renilla luciferase activity, relative to that of the control (no heat shock and no riluzole; the ratio of firefly/Renilla set at 1). Panels (A) and (B) are the average±SD of the result from four independent experiments each with 4 separate determinations. Panels (C) and (D) are the result from one single experiment with four different sample/determinations. Panels (A) and (C) show results on the reporter gene under both basal (37°C) and heat shock (42°C, 2 hr) condition; panels (B) and (D) show the effect of riluzole on the basal luciferase reporter gene activity on an expanded Y-axis. * and ** denotes, respectively, two-tailed t-test with a probability of difference between 0.01–0.05 (significant) and <0.01 (highly significant).

The effects of riluzole on the basal (control; 37°C) and heat shock-induced (42°) hsp70-luciferase reporter gene activity in the human HeLa cell line is shown in Fig. 1. Fig. 1A and 1B represent the average±standard deviation of four separate experiments each with 4 separate determinations, and Fig. 1C and D is the average±standard deviation of four separate determinations from one experiment. We show in fig. 1A that expression of the hsp70-reporter was induced 36 fold on average by heat shock. Pre-incubation of the cells with riluzole for 16 hr followed by heat shock gave a riluzole dose-dependent amplification of the heat shock induction of hsp70-firefly luciferase reporter gene expression (solid symbol, Fig. 1A); at the optimal riluzole concentration of 1–2 μM, the heat shock induced hsp70-reporter gene activity was ∼2.7×higher than that of heat shock control (without riluzole). Analysis of the effects of riluzole on the basal (i.e. 37°C) expression of hsp70-luciferase as shown in Fig. 1B revealed a qualitatively similar effect: both in terms of the optimal concentration of riluzole (1–2 μM) and the fold of enhancement (2.5–2.8 fold without/with riluzole). The large standard deviation of the heat induced reporter gene activity shown in Fig. 1A (solid symbol) is due to experiment-to-experiment variation in the basal (37°C) reporter gene activity and this translates to a wide range in the fold of induction by heat shock (e.g. in the absence of riluzole, the range of heat shock induction was 15 to 71 fold over that of the 37°C control for the four different experiments in Fig. 1A). Similar variation in the basal HSF1 activity under normal conditions has previously been noted [16]. Within a given experiment using the same pool of transfected cell, however, the sample-to-sample variation was <10% (Fig. 1C and 1D). This pattern was consistently observed throughout the many experiments done over a two-year period. The effect of riluzole in amplifying the hsp70-reporter gene expression required a pre-incubation period: the addition of riluzole at or within 1–2 hr of heat shock (before or after) had little effect (data not shown).

The ability of riluzole to amplify hsp70-reporter gene expression is not limited to the HeLa cells. Similar and reproducible results were obtained using a variety of other human and rodent cell lines and primary cell cultures including immortalized striatal neurons of the ST14D2 and N548 lineages [17] as well as primary cultures of embryonic CNS neurons. Because of the wealth of literature information on the regulation and function of HSF1 and the robust transcriptional regulation of heat shock gene expression in HeLa cells-information that provided a solid backdrop for our studies on the effects of riluzole in modulating the HSR and in regulating the amount and activity of HSF1–many of the experiments presented in this study were done using HeLa cells. Results on the effect of riluzole on hsp70-reporter gene expression and survival of primary embryonic spinal cord neurons are also presented in this study.

### Effects of riluzole on the regulation and function of HSF1

**Figure 2 pone-0002864-g002:**
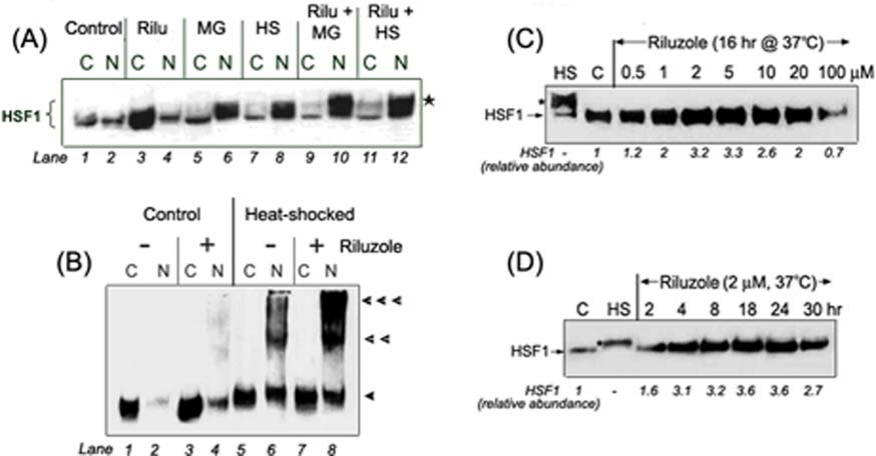
Effects of riluzole on the amount, distribution and trimerization of HSF1. (A) Effects of riluzole, MG132 and heat shock on the amount and distribution of HSF1 in the cytosol and nuclear fractions. Post-confluent HeLa cells in 60 mm plates were used. Riluzole (Ril) was added to designated plates to a final concentration of 1.5 μM and incubated at 37°C for 18 hr. For treatment with MG132 (MG), a proteasome inhibitor, it was added to designate plates to a final concentration of 5 μM and incubated at 37°C for 3 hr. Condition for heat shock was 42°C for 2 hr prior to harvesting of cells. When riluzole was used in combination with MG132 or heat shock, cells were treated with 1.5 μM riluzole for a total of 18 hr; MG132 was added during the last 3 hr or heat shocked at 42°C for the last two hrs. The cytosol (C) and nuclear (N) fractions were prepared as described [22]. Aliquots containing 10 μg protein were loaded onto 8% SDS-polyacrylamide gel for immuno-Western blot probing of HSF1. The position of the hyperphosphorylated HSF1 is indicated by *. (B) Effects of riluzole and heat shock on the distribution and trimerization of HSF1. Control and riluzole (1.5 μM; 16 hr) treated cells were incubated under control (37°C; lanes 1–4) and heat shocked (42°C, 1 hr; lanes 5–8) conditions. Cells were harvested and cytosol (C) and nuclear (N) fractions prepared [22]. To assess the stoichiometry of HSF1, proteins were crosslinked with 2 mM glutaraldehyde at room temperature for 10 min followed by quenching of the crosslinking reaction with the addition of 100 mM lysine. Samples were subjected to SDS-PAGE (4–12% acrylamide gel) followed by immuno-Western blot detection of HSF1. The single, double, and triple arrow heads indicate, respectively, the positions on the gel of the HSF1 monomer, dimer, and trimer. (C) Dose response effect of riluzole on the accumulation of HSF1. Cells were incubated with concentrations of riluzole from 0.5, 1, 2, 5, 10, 20 and 100 μM for 16 hr at 37°C. Cells were harvested and aliquots of the whole cell extracts containing 10 μg protein were used for immuno-Western blot detection of HSF1. The relative abundance of the HSF1 signal determined by densitometry is presented at the bottom of the figure. Samples from heat shocked (42°C, 2 hr) and control cells served as controls. The hyperphosphorylated form of HSF1 is indicated by an *. (D) Time course of effect of riluzole treatment on the steady state level of HSF1. Cells were treated with 2 μM riluzole at 37°C for time periods indicated (2, 4, 8, 24 and 30 hr). Cells were harvested and aliquots of the whole cell extracts containing 10 μg protein were used for immuno-Western blot detection of HSF1. The relative abundance of the HSF1 signal determined by densitometry is presented at the bottom of the figure. Samples from heat shocked (42°C, 2 hr) and control cells served as controls. The hyperphosphorylated form of HSF1 is indicated by an *.

HSF1 mediates the HSR: stress acutely converts the constitutively expressed dormant, monomeric HSF1 in the cytosol to a nuclear localized, hyperphosphorylated HSF1 trimer that binds and trans-activates the heat shock promoters [18]. In order to gain a better understanding of the mechanism by which riluzole enhanced the hsp70-luciferase reporter gene expression shown in Fig. 1, we used immuno-Western blot techniques to evaluate the effects of riluzole on the amount, distribution (cytosol versus nuclear compartment), and trimerization of HSF1. The effects of the proteasome inhibitior, MG132, and heat shock–known activators of HSF1–were included as positive controls in the experiment [19], [20], [21]. We show in Fig. 2A that HSF1 was detected in both the cytosol and nuclear fractions of control HeLa cells (lanes 1 and 2); the relative distribution of HSF1 in cytosol versus nucleus varied between experiments depending on the cell culture condition (compare lanes 1&2 of Fig. 2A versus 2B,), and this is consistent with the wide range of HSF1 DNA-binding activity observed under normal conditions [16] and with the result of our reporter gene assay (Fig. 1). Treatment of HeLa cells with riluzole (1.5 μM, 18 hr; lanes 3 and 4) gave a significant increase in the amount of HSF1 in the cytosol and a small but reproducible increase in the nuclear fraction (Fig. 2A: cytosol, lanes 3 versus 1; nuclear, lanes 4 versus 2). This is to be contrasted with the effects of proteasome inhibition (MG132, 5 μM, 3 hr; lanes 5 and 6) and heat shock (42°C, 2 hr; lanes 7 and 8) that acutely promoted the activation of HSF1- as indicated by nuclear localization and hyperphosphorylation (supershift of the HSF1 band). Pre-treatment of the cells with riluzole followed by either MG132 (5 μM, 37°C for last 3 hr) or heat shock (42°C for the last 2 hr) gave a greater mobilization and accumulation of the HSF1 in the nuclear compartment (lanes 9–12). The result in Fig. 2A suggests that riluzole increased the amount of HSF1 principally in the cysotol, and this increased reserve allowed for a greater mobilization and nuclear translocation of HSF1 upon stress. To validate this possibility, we use a protein-crosslinking technique in conjunction with immuno-Western blot to determine the amount and the stoichiometry of HSF1 [22]. We show in Fig. 2B that riluzole increased the amount of monomeric HSF1 in the cytosol of control, unstressed cells (Fig. 2B: lane 3 versus 1). Riluzole also had a small and reproducible effect in increasing the amount and trimerization of HSF1 in the nuclear compartment of cells maintained at 37°C (Fig. 2B: lanes 4 versus 2). Heat shock promoted the nuclear translocation and trimerization of HSF1, and these effects of heat shock were amplified by the pre-treatment of cells with riluzole (Fig. 2B: lanes 8 versus 6). The effect of riluzole was time and dose dependent: we show in Fig. 2C and D that an optimal increase in the amount of HSF1 was observed after ∼18 hr of incubation with ∼2 μM of riluzole.

**Figure 3 pone-0002864-g003:**
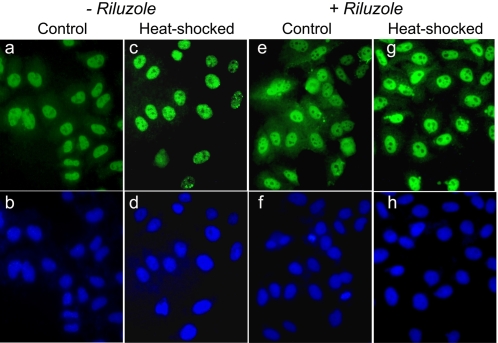
HSF1 immuno-fluorescence photomicrographs of control and heat shocked HeLa cells without and with riluzole pre-treatment. Riluzole (2 μM) was added to HeLa cells and incubated at 37°C for 16 hr (panels e–h). Designated plates of cells were heat shocked at 42°C for 2 hr (panels c, d, g, h). Control cells were incubated at 37° for equivalent time periods. Cells were fixed, permeabilized and stained for HSF1 according to methods described in the text (panels a, c, e and g). Nuclei were counter stained with Hoechst 33342 (panels b, d, f and h). Images of representative fields were captured with a SPOT camera system (Diagnostic Instruments, Inc., Sterling Heights, MI).

We also used immunocytochemical staining techniques to evaluate the effects of heat shock and riluzole treatment on the distribution and amount of HSF1. We show in Fig. 3 that HSF1 had a diffuse cytoplasmic and nuclear distribution in unstressed cells (panel a). Heat shock promoted the nuclear localization of HSF1 (panel c). Riluzole increased the diffused HSF1 staining intensity in both the cytosol and nucleus (panel e), and the pretreatment with riluzole followed by heat shock gave a greater increase in the nuclear HSF1 when compared to heat shock alone although there remained some diffuse staining in the cytosol (panel g).

### The synergistic cytoprotective activity of riluzole and conditioning heat shock

**Figure 4:**
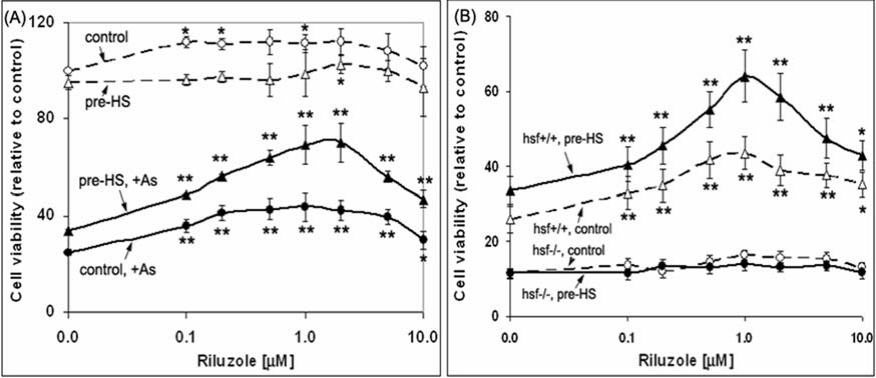
Synergistic effects of riluzle and conditioning heat shock in conferring cell survival under oxidative stress requires a functional HSF1. (A) Dose response effect of riluzole and conditioning heat shock on cell viability in the absence and presence of oxidative stress challenge. HeLa cells in 96 Strip-well plate were used. The conditions for riluzole treatment and conditioning heat shock were as described in the text. To test for cell survival under conditions of oxidative stress, 20 μM sodium arsenite was added and incubated at 37°C for 24 hr. Viability of the cells was determined usin*g the* CellTiter-Glo luminescent reagent from Promega Inc. Cell viability signal, relative to that of the untreated control, is plotted as a function of the concentration of riluzole added. Result represents the average of four independent determinations±standard deviation. * and ** denotes, respectively, two-tailed t-test with a probability of difference between 0.01–0.05 (significant) and <0.01 (highly significant) of the riluzole-treated samples from that of the minus riluzole control. (B) The cytoprotective activity of riluzole and conditioning heat shock requires a functional HSF1 protein. Murine embryo fibroblasts derived from hsf1−/− knockout mice [25] and its hsf1+/+ normal littermate were plated in 96 a Stripwell plate. The conditions used from the treatment of cells with riluzole, conditioning heat shock at 42°C for 2 hr, and assessment of the “cell-kill” effects of arsenite were as described in the text. The figure presents data on viability of the arsenite-challenged cells pretreated with various concentrations of riluzole, without and with conditioning heat shock. Data on viability of the control cells (i.e. without arsenic challenge) are not included in Fig. 4B as they were qualitatively similar to that of the HeLa cells shown in Fig. 4A.

Induction of the HSPs provides an important cytoprotective mechanism for survival under stress. In this context, our observation that riluzole increased HSF1 reserve to allow for a more robust mobilization of HSF1 and induction of the HSR would suggest that riluzole and conditioning heat shock could have synergistic effect in protecting cells against stress-induced injury and death. In Fig. 4A, we show the dose-dependent effects of riluzole and conditioning heat shock on cell viability in the absence and presence of sodium arsenite-induced oxidative stress challenge [23], [24]. Cell viability was expressed as a % of that of the control (no riluzole, no heat shock, no arsenite). We showed that: (1) arsenite (20 μM, 24 hr) by itself decreased cell viability by 75%; this cytotoxic effect was countered somewhat by the pretreatment of cells with riluzole with an optimal protection observed at 1 μM (viability 25 & 45% for 0 and 1 μM riluzole, respectively; solid circle, solid line). (2) Conditioning heat shock (pre-HS) increase cell survival from 25 to 34% in the arsenite-challenged cells. Further, the treatment of cells with riluzole followed by conditioning heat shock had a synergistic effect in promoting cell survival when challenged with arsenite (solid triangle, solid line); at 1 μM riluzole, the percentage of viable cells was 75 versus 45%, with and without conditioning heat shock, respectively. (3) In the un-challenged cells (no arsenite): riluzole by itself had a small but reproducible effect in promoting cell growth/viability (open circle), whereas conditioning heat shock reduced cell growth/viability by ∼10% (open triangle).

In order to validate that the effects of riluzole and conditioning heat shock to confer protection for survival under stress are related to their effects on the regulation and function of HSF1, a parallel experiment was done using hsf1−/− and their wild-type counterpart hsf1+/+ murine embryo fibroblasts [25], [26]. The result on cell viability of the arsenite-challenged cells is shown in 4B. The cell viability profile of the hsf+/+ MEF (triangle symbol) was similar to that of the HeLa cells shown in Fig. 4A–that riluzole and conditioning heat shock had synergistic effects in promoting survival when challenged with arsenite. The hsf−/− MEF, by comparison, was more sensitivity towards the cytotoxic effects of arsenite, such that ∼10% of the hsf−/− cells survived the aresenite challenge as compared to ∼25% of the hsf1+/+ cells. Importantly, pre-treatment of cells with riluzole followed by conditioning heat shock had little or no effect in enhancing survival of the hsf1−/− MEF (solid and filled circle symbols). This result underscores the critical role of HSF1, and the presumptive induction of HSPs in conferring cell survival under stress. Together, the results of Fig. 1–4 show that riluzole increased the amount of HSF1 to afford a most robust HSR for survival under stress.

### Riluzole amplifies the HSR and protects spinal cord neurons against oxidative stress induced death

**Figure 5:**
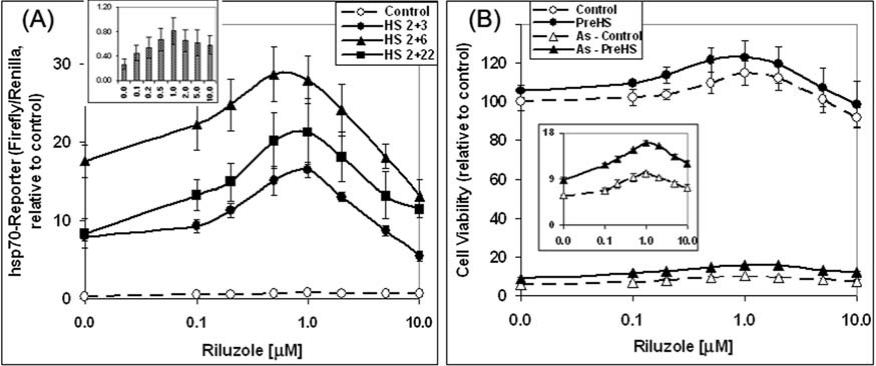
Riluzole amplifies the heat shock response and enhances survival under oxidative challenge in embryonic spinal cord neurons. (A) Dose-response effect of riluzole on the basal and heat-induced hsp70-luciferase reporter gene in spinal cord neurons. Cells were transfected with the hsp70-firefly luciferase and Renilla luciferase DNA at 10 days in vitro (DIV), a time when the neurons were fully differentiated with a meshwork of processes. Cells in 96 well plates were treated with specified concentrations of riluzole at 37°C for 16 hr. Cells were then heat shocked at 42°C for 2 hr followed by recovery at 37°C for 3, 6 and 22 hr. Cells were harvested and processed for reporter gene assay according to methods described in the text. Result represents the average of four independent determinations±standard deviation. The insert shows an expanded scale of reporter gene activity to better illustrate the effect of riluzole on the basal level (37°C) of reporter gene expression. (B) Synergistic effects of riluzole and conditioning heat shock to enhance survival of spinal cord neurons under oxidative challenge. The conditions of riluzole treatment, heat shock, and arsenite-challenge were as described in the text (same as protocol used in the experiment shown in Fig. 4A for NG108-15 cells). Viability of the cells was determined usin*g* the CellTiter-Glo luminescent reagent from Promega Inc. Cell viability signal, relative to that of the untreated control (line 1; without riluzole, 100%), is plotted as a function of the concentration of riluzole added. The insert is an expanded scale to better illustrate the fraction of cells that survived the arsenite-induced oxidative challenge. Result represents the average of four independent determinations±standard deviation.

ALS is a motor neuron disease with symptoms and disease progression resulting from degeneration and death of motor neurons in the spinal cord and other CNS regions [27]. The cause of motor neuron death in ALS is not entirely clear and likely involves a multitude of related contributing factors including glutamate excitotoxicity, oxidative stress, and protein mis-folding and aggregation [28], [29], [30]. In Fig. 5, we evaluated the effects of riluzole on induction of the hsp70-reporter gene activity and in conferring survival in primary cultures of embryonic spinal cord.

Embryonic spinal cord neurons were transfected with the hsp70-firefly luciferase and Renilla luciferase DNA DNA at 10 DIV. The effects of heat shock and of the effect of pre-treatment with riluzole on hsp70-reporter gene expression is shown in Fig. 5A. Heat shock increased the hsp70-reporter gene expression in primary cultures of embryonic spinal cord by ∼5–20 fold–a magnitude that is significantly lower than what was observed in the HeLa cells in Fig. 1. This observation is consistent with result of previous studies: spinal cord neurons have a high threshold for induction of the HSR both in terms of the severity of stress and the magnitude of induction [23], [24], [31], and induction of the HSR is inversely proportional to the “differentiation” state of neuronal cells [23], [24]. Pre-treatment of the spinal cord neuron culture with riluzole gave a dose-dependent amplification of the heat shock induction of hsp70-reporter gene activity. In Fig. 5A, this effect of riluzole was illustrated using three different protocols of heat shock and recovery: heat shock for 2 hr followed by recovery for 3 hr (filled circle), 6 hr (filled triangle), and 22 hrs (filled square). In each case, an optimal enhancement was observed at 0.5–1 μM riluzole, and the protocol of heat shock for 2 hr followed by recovery for 6 hr gave the greatest increase in reporter gene activity. Shown as an insert in Fig. 5A is an expanded scale of the basal 37°C reporter gene activity to show that riluzole also increased the basal hsp70-reporter gene activity.

In Fig. 5B, we show that riluzole and conditioning heat shock increased cell survival under conditions of arsenite-induced oxidative stress, an effect that can be correlated with the enhanced HSR shown in Fig. 5A. We note that embryonic spinal cord neurons appear to be exquisitely sensitive to the cytotoxic effects of arsenite, such that a 24 hr incubation with 20 μM of arsenite resulted in ∼95% cell kill with 5% cell survival (see insert of Fig. 5B). Riluzole at 1 μM increased cell survival to 10%. Conditioning heat shock by itself prior to arsenite challenge increased survival from 5 to 8%. Riluzole plus conditioning heat shock had a synergistic effect: at the optimal concentration of 1 μM of riluzole, cell survival was increased to 16%.

### Riluzole slows the turnover of HSF1

**Figure 6:**
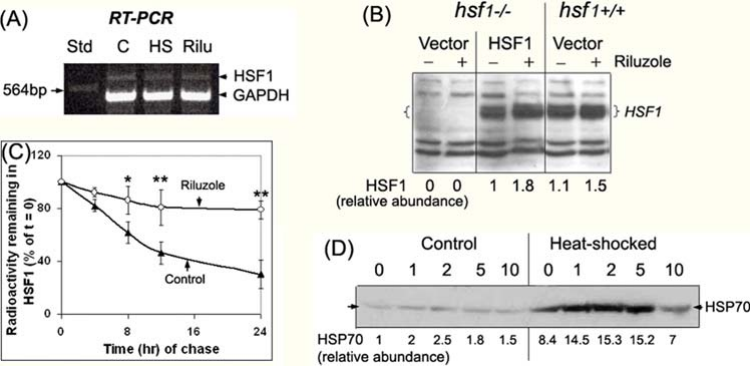
Effects of riluzole on the expression, turnover and activity of HSF1. (A) Reverse-transcriptase PCR analysis of the effects of heat shock and riluzole treatment on the expression of mRNA^hsf1^. RNA was isolated from control, heat shocked (2 hr, 42°C), and riluzole-treated (2 μM, 8 hr) HeLa cells. RNA was reverse transcribed and PCR-amplified using HSF1-specific primers. Std: a 564 bp fragment from *Hind*III digest of λ DNA. The positions of the 642 bp HSF1 DNA fragment and the co-amplified 481 bp GAPDH internal control are as indicated. (B) Effects of riluzole on the endogenous versus CMV-promoter driven HSF1 expression. An episomal eukaryotic expression vector of the human HSF1, pCep4hHSF1, was used to drive the expression of HSF1 in hsf1−/− MEF. Cells were allowed to recover at 37°C for 6 hr after DNA transfection. Riluzole was then added to designated plates to a final concentration of 2 μM and incubated at 37°C for 16 hr. Cells were harvested and RIPA extracts prepared. Control and riluzole-treated hsf1−/− cells transfected with the pCep4 vector as well as hsf+/+ cells were included as controls in the experiment. Result on the relative abundance of HSF1 is shown at the bottom of the figure. (C) Pulse-chase analysis of the turnover of HSF1 in control versus riluzole-treated HeLa cells. Cells were labeled with [^35^S]methionine as described in the text. At the end of this labeling period, cells were rinsed extensively the chase initiated either without (Control, solid symbol) or with 2 μM riluzole at 37°C (open symbol). Samples were harvested at 0, 4, 8, 12 and 24 hr after initiation of the chase. Aliquots of the cell extracts were used for immunoprecipitation of HSF1 using protein A for pull-down by centrifugation. Result on the amount of radioactivity remaining with the HSF1 immunoprecipitate, relative to that of the t = 0 chase control, is plotted against the time of chase. Result is the average of 4 separate determinations±standard deviation. The single and double asterisk symbols, * and **, indicate two tailed t-test of the control vs. riluzole-treated samples with probability of difference of 0.01–0.05 (*, significant) and <0.01 (**, highly significant), respectively. (D) Dose-response effect of riluzole on HSP70 expression under control and heat-shocked conditions. HeLa cells were treated with concentrations of riluzole as indicated for a total of 18 hrs. For heat shock, cells, at 10 hrs after the addition of riluzole, were placed in a 42°C incubator for 2 hr followed by recovery at 37°C for 6 hr. Aliquots of the RIPA cell extract containing 10 μg protein were used for immuno-Western blot analyses of HSP70 [23]. The relative abundance of HSP70 is indicated at the bottom of the figure.

To better understand how riluzole acts to increase the amount of latent HSF1, we used reverse transcriptase-polymerase chain reaction (RT-PCR) to assess the effects of heat shock and riluzole treatment on the abundance of mRNA^hsf1^ using HeLa cells as a model for these studies. In the experiment of Fig. 6A, a 642 bp HSF1 fragment was co-amplified with a 481 bp fragment of glyceraldehyde-3-phosphate dehydrogenase (GAPDH) using appropriate primers. We show that neither heat shock nor riluzole treatment had a significant effect on the RT-PCR product of either HSF1 or GAPDH. To assess if the effects of riluzole is transcriptional and thus promoter specific, we transfected hsf−/− MEF [25], [26] with the episomal eukaryotic expression vector of hsf1-pCep4hHSF1-to force the expression of HSF1 from the CMV early enhancer/promoter and determined the effects of riluzole on this expression. The experiment in Fig. 6B showed that hsf−/− cells transfected with the empty pCep4 vector gave no detectable HSF1 signal (lanes 1 and 2). Transfection of the cells with pCep4hHSF1 drove the expression of HSF1, and riluzole treatment increased the level of HSF1 (lane 3 & 4). Treatment of the hsf+/+ cells with riluzole gave a similar increase in HSF1 (lanes 5 & 6). Analyses of the effects of riluzole on the turnover of HSF1 by pulse-chase and immuno-precipitation of HSF1 in Fig. 6C shows that riluzole slowed the rate of decay of [^35^S]methionine-labeled HSF1 during chase, suggesting that riluzole slowed the turnover of the HSF1 protein to effect an increase in the steady state level of the protein. The fractional rate (Kd) of HSF1 was calculated to be 0.051 and 0.01/hr for the control and riluzole-treated samples, and this translated to HSF1 half-lives estimates of 13.6 and 69.3 hr, respectively. In Fig. 6D, we show that the riluzole-induced increase in HSF1 drove a higher expression of the 72 kDa HSP70 protein under both control (37°C) and heat shock (42°C) conditions, with an optimal increase observed at 2 μM riluzole.

Protein degradation in the eukaryotic cells primarily involves the ubiquitin proteasome (UPS) and the lysosomal systems. There is ample literature evidence that inhibition of the UPS pathway, e.g. by the use of peptide aldehyde inhibitors including MG132, promotes the rapid activation of HSF1 [19], [20], [21]. Our result on the effects of MG132 on HSF1 in Fig. 2A is consistent with these published observations. Further, other proteasome inhibitors including epoxomicin, YU102, Ac-Ala-ProlNle-Asp-al; clasto-lactacystin β-lactone; and Ada-(ahx)3-(Leu)3-vinyl sulfone (BIOMOL Int, LP) also promoted the activation of HSF1 as determined by it's hyperphosphorylation, nuclear translocation and trimerization (data not shown). The effect of riluzole was qualitatively different from drugs that inhibit proteasome function: riluzole gave a delayed increase in the amount but not the acute activation of HSF1.

**Figure 7:**
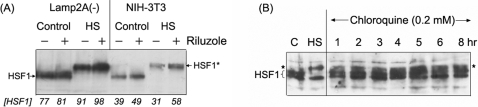
Consequences of genetic and pharmacological blockade of protein degradation pathways on the regulation of HSF1. (A) Effects of riluzole on HSF1 in NIH-3T3 versus Lamp2A RNAi-knockdown cells. Cells in 60 mm plates were treated with 2 μM riluzole at 37°C for 16 hr. For heat shock, cells were placed in a 42°C incubator for 2 hrs. Cells were harvested and aliquots of the RIPA cell extracts containing 10 μg protein were used for immuno-Western blot analysis of HSF1 according to methods described in the text. The position on the gel of the HSF1 and of the heat induced hyperphosphorylated HSF1 is indicated by an *. The relative abundance of the HSF1 protein is indicated at the bottom of the figure. (B) Effects of chloroquine on HSF1. HeLa cells were treated with 0.2 mM chloroquine at 37°C for time periods as indicated. Aliquots of the RIPA cell extract were used for immuno-Western blot detection of HSF1. Extracts from control and heat-shocked cells were included as controls. The position on the gel of the HSF1 and of the heat induced hyperphosphorylated HSF1 is indicated by an *.

There are at least three different lysosomal mechanisms of protein degradation: autophagy (aka, macroautophagy, MA), chaperone-mediated autophagy (CMA), and micro-autophagy pathways. Macro-autophagy is considered a non-selective process of “self-eating” involving the formation of intracellular biomembrane that sequester a portion of the cytosol and whole organelle to form the autophagosome. Chaperone-mediated autophagy, on the other hand, requires the binding of specific substrate protein to the constitutively expressed HSC70 cognate protein and binding of the complex to the lysosomal receptor Lamp2A for the importation and intra-lysosomal degradation of the substrate. We showed in Fig. 7 that whereas riluzole up-regulated the expression of HSF1 of both the control and heat-shocked NIH-3T3 cells, riluzole had little effect in the Lamp2A(-) RNAi knock-down cells [32], [33]. The effect of riluzole is distinct from that of lysozomotropic agents such as chloroquine or ammonium chloride. We show in Fig. 7B that chloroquine (0.2 mM) gave a time-dependent increase in the hyperphosphorylation (supershift in gel) of HSF1. Further, in despite the activation of HSF1, chloroquine treatment had dire consequence in cell viability–most of the cells were dying/dead after 8 hr incubation in the presence of 0.2 mM chloroquine. These effects of chloroquine are qualitatively different from that of riluzole.

## Discussion

Induction of the HSR provides a ubiquitous and important cytoprotective mechanism. A major obstacle in harnessing such cytoprotective activity for therapeutic purposes is that agents/conditions that induce the HSR are proteotoxic, and the induction of HSPs under such conditions represents a compensatory mechanism to rectify the perturbation of protein homeostasis. We used a cell-based, hsp70-luciferase reporter gene assay in a 96 well format to identify drugs/small molecules that are not by themselves robust inducers of the HSR but which can enhance/amplify the effects of HSR inducers. We show here that the FDA-approved ALS drug riluzole slows the turnover of HSF1 to effect a more robust HSR for cytoprotection. These effects of riluzole appear to be ubiquitous in a variety of cell types/lines that we studied including the human HeLa cells–which we used as a model to evaluate the effects of riluzole on the regulation and function of HSF1, and spinal cord neurons–which we used to validate the relevance of these effects of riluzole. Our result provides novel insight into the mechanism of turnover of HSF1, and identifies the degradation of HSF1 as a target for therapeutics intervention.

Studies on the effects of riluzole on hsp70-luciferase reporter gene show that riluzole increased both the basal (37°C) and the heat shock (42°C)-induced hsp70-reporter gene, such that the fold of heat shock induction (i.e. HS/control) remained relatively constant in the absence and presence of various concentrations of riluzole. Analysis of the effects of riluzole on HSF1 by immuno-Western blot and cytochemistry techniques showed that riluzole markedly increased the amount of latent, cytosolic HSF1 monomer; this increased reserve allowed for a greater mobilization of HSF1 upon heat shock. We further note that riluzole by itself had a smaller but reproducible effect in increasing the amount of nuclear localized, activated HSF1 trimer–an effect likely secondary to the increased cytosolic HSF1. Indeed, spontaneous activation of HSF1 is often observed upon over expression of HSF1 in transfected mammalian cells [16], [34], [35], [36], and recombinant HSF1 expressed in E. coli is in a constitutively active oligomeric form [18], [37], [38]. These considerations suggest that both the basal (37°C) and the heat shock-induced (42°C) hsp70-reporter gene activity is an accurate and valid assessment of the activity of HSF1-an activity which includes the trimerization and nuclear translocation of HSF1 and binding of the HSF1 trimer to the heat shock element to transactivate the hsp70 promoter [18]. The notion that the basal reporter gene activity is in fact HSF1-dependent is further support by the absence of a measurable activity when the hsp70-luciferase DNA is transfected into hsf−/−cells (data not shown).

Our conclusion that riluzole increased the amount of HSF1 to afford a more robust HSR for cytoprotection is supported by three lines of evidence reported in this study: (1) Riluzole and conditioning heat shock had synergistic effects in protecting cells against the cytotoxic effects of arsenite-induced oxidative stress. (2) Riluzole gave similar, if not identical, dose-response profiles in increasing the amount of HSF1 (Fig. 2), in enhancing hsp70-reporter gene expression (Fig. 1 and 5), in promoting the expression of HSP70 (Fig. 5), and in conferring cytoprotection (Fig. 3 and 5); such correlation is consistent with a cause-effect relationships of the increased HSF1, enhanced induction of HSPs, and improved cell survival. (3) Genetic deletion of hsf1 negated the cytoprotective effects of both riluzole treatment and of conditioning heat shock.

Riluzole slowed the turnover of HSF1 via the CMA pathway of protein degradation as RNAi-mediated knockdown of Lamp2A, an essential lysosomal membrane receptor protein involved in CMA, blunted the effect of riluzole. Most substrates for CMA contains a KFERQ-like sequence, a binding motif to the cytosolic HSC70 protein and targeting of the protein complex to the Lamp-2A receptor located on the cytosolic face of the lysosomal membrane [39]. Analysis of both the human and mouse HSF1 failed to reveal an idealized KFERQ sequence, however, it is well known that HSF1 interacts with both the HSC and HSP70 proteins *in vivo* and such interaction modulates the activity and stability of HSF1 [40], [41], [42], [43], [44]. CMA is active in most cell types and is activated under conditions of nutrient deprivation [39]. Consistent with this, we note that the effect of riluzole is not cell type specific and is more pronounce in nutrient deprived post-confluent cells (unpublished observations). At this time, we do not know if riluzole targets a step in the CMA pathway or that it targets the HSF1 protein as a substrate for this pathway. If it is the former, one would expect riluzole to block the CMA-mediated degradation of known substrates such as glyceraldehyde-3-phosphate dehydrogenase and RNase [32], [33]. If riluzole targets HSF1, perhaps by modulating a post-translation modification event of HSF1 necessary for its degradation via CMA, then the effect of riluzole is likely to be more specific and limited to HSF1 and perhaps other select CMA-substrates. Analysis of the effects of riluzole in an *in vitro* CMA-mediated protein degradation system should help to provide some answers to these questions [32], [33].

The effect of riluzole on HSF1 is qualitatively different from that of MG132 and chloroquine, inhibitors of the proteasome and lysosome, respectively; they promoted the hyperphosphorylation, nuclear translocation and activation of HSF1. Their mechanisms of action likely involve the blockage of bulk protein degradation, leading to the buildup of abnormal, mis-folded proteins and consequently activation of the HSR. It is well known that the accumulation of abnormal proteins in cells, either due to genetic mutation or by the artificial introduction of denatured proteins, will trigger the HSR [45], [46]. We further note that the effect of riluzole was unaffected by genetic deletion of Atg5, an essential gene for the macro-autophagy pathway of protein degradation [47]: riluzole up-regulated the expression of HSF1 in both the wild type and the atg5−/− knockout murine embryo fibroblasts (unpublished observation).

Our working hypothesis on the regulation of HSF1 by riluzole is: (1) Normal turnover of HSF1 likely involves, at least in part, the CMA pathway: cytosolic HSF1 monomer associates with HSC/HSP70 proteins and the complex is targeted for degradation by CMA. (2) Riluzole, by mechanisms to be delineated, inhibits this degradation leading to an increased accumulation of HSF1. (3) The increased HSF1 reserve affords a greater mobilization of HSF1 under stress for a more robust induction of the HSP chaperones to confer cytoprotection.

Other drugs have been shown to modulate the HSR. In particular non-steroidal anti-inflammatory drugs (NSAIDS) are co-inducers of the HSR: they partially activate components of the HSR and often work in conjunction with a secondary stress signal for full induction of HSP70 expression [6], [48]. Several clinical stage hydroxylamine derivative compounds, arimoclomol, iroxanadine and bimoclomol, are also co-inducers of the HSR: they promote the hyperphosphorylation and prolong the activation of HSF1 to enhance the production of HSPs following heat shock [49], [50], [51], [52], [53]. Treatment of transgenic ALS mice with arimoclomol improves behavioral phenotypes, prevents neuronal loss and extends survival rates of the ALS mice by 22%.

The effect of riluzole on the HSR is mechanistically distinct from the co-inducers: riluzole up-regulates HSF1 reserve to function as an amplifier of the HSR. We believe that riluzole may be a prototype of drugs that can amplify the HSR for cytoprotection, and we will continue to use our cell-based reporter gene assay to screen for and identify such compounds. We further note that the recommended dose for ALS is one 50-mg Rilutek® tablet (MW 234.2; Aventis Pharmaceuticals) every 12 hrs, and patients have continued on the treatment regimen for up to 5 years. Assuming an average body weight of 85 kg of the adult male, body water content of 60% of weight, and average absolute oral bioavailability of about 60% (http://products.sanofi-aventis.us/rilutek/rilutek.html), this would translate to a circulating concentration of ∼5 μM riluzole. The similarity in the range of bioactive concentrations of riluzole in patients vs. our cell-based *in vitro* system is remarkable and reassuring.

We suggest that drugs that target the amount/turnover of HSF1 may provide a means for therapeutic intervention in protein mis-folding diseases. While riluzole is the first and only FDA-approved drug for ALS, its clinical efficacy is limited–it extends time to death/tracheostomy by ∼3 months (http://products.sanofi-aventis.us/rilutek/rilutek.html). Our work described here provides a framework to evaluate the possible synergistic cytoprotective effects of riluzole and small molecule elicitor(s) of the HSR [6].

## Materials and Methods

### Materials

Riluzole was obtained from Sigma Chem. Co. and AB Chem Tech. Both sources of riluzole gave similar results. Our RTG88 rabbit polyclonal anti-hHSF1 antibody was prepared by immunizing a rabbit with histidine-tagged human HSF1 produced in *E. coli* and purified by affinity chromatography using Ni-NTA resin from Qiagen; the purified protein was sent to Cocalico Biologicals, Inc. for the purpose of antibody production. The RTG-88 antibody was similar to that of the SPA-901 anti-HSF1 antibody from Stressgen/Assay Designs, although RTG88 appeared to be more specific and superior in sensitivity and gave little or no background when used to detect the human HSF1 protein. For detection of HSF1 in rodent cells (NIH-3T3 and Lamp2A−/−), a rabbit polyclonal antibody (#4356) from Cell Signaling Tech. was used. Immuno-Western blot detection and quantitation of the heat inducible 72 kDa HSP70 protein was done using a rabbit polyclonal antibody from Stressgen (SPA812). The pCep4 episomal eukaryotic expression vector, lipofectamine 2000 reagent used for DNA transfection, and RT-PCR reagents were from Invitrogen Co. The humanized Renilla DNA (phRLSV40), Dual-Glo luciferase assay reagent (E2920), and the CellTiter-Glo luminescent cell viability assay reagent (G7571) were from Promega Inc. Proteasome inhibitor pack (PW9900) that include MG132, epoxomicin, YU102, Ac-Ala-ProlNle-Asp-al; clasto-lactacystin β-lactone; and Ada-(ahx)3-(Leu)3-vinyl sulfone were from BIOMOL Int, LP. RIPA (RadioImmunoPrecipitation Assay) buffer used for cell extract preparation had the following composition: 150 mM NaCl; 10 mM Tris, pH 7.2; 0.1% SDS; 1.0% Triton X-100; 1% deoxycholate; 5 mM EDTA; 1 mM phenylmethylsulfonyl fluoride; 2 μg/ml leupeptin; 100 μM sodium orthovanadate). All other biochemical and chemicals were of molecular biology or reagent grade.

### Cell culture and conditions of riluzole treatment and heat shock

HeLa cells were grown in Dulbecco's Modified Eagle's Medium (Mediatech Inc.) supplemented with 10% fetal bovine serum, 50 μg/ml streptomycin and 50 U/ml of penicillin. Cells were subcultured at or near confluency by minimal trypsinization (0.25% trypsin; Mediatech Inc.) and dispersion into single cell suspension in new growth medium and plating onto new growing surfaces. Cells were allowed to grow to confluency and unless indicated otherwise, experiments were done using post-confluent cells. For riluzole treatment, a 100 mM stock solution was diluted with DMEM to a 10× working stock and added to the cell culture medium and incubated at 37°C for time indicated. The hsf1−/− and the wild type hsf1+/+ murine embryo fibroblasts (McMillan et al., 1998) and the NIH3T3 and the RNAi knock down Lamp2A cells (Massey et al., 2006) were cultured in DMEM under standard conditions.

Primary cultures of embryonic spinal cord neuron were prepared essentially as previously described [54]. Briefly, the spinal cords were dissected from embryonic day 16 (E16) rat embryos. Meninges were removed, and the cords were dissociated with gentle trituration. Cells were plated at a density of 350 neurons/mm^2^. The mixed cultures were grown in serum-containing medium (89.4% Minimum Essential Medium, 10% horse serum, 0.6% glucose, supplemented with penicillin and streptomycin) for 6 days at 37°C and 5% CO2 before treatment. To enrich for neurons, the serum containing medium was changed to Neurobasal medium (Gibco/Invitrogen) supplemented with B-27, penicillin, and streptomycin at 24 h after plating (1 day in vitro, DIV). After an additional 24 h, cytosine arabinoside (Ara-C, 5 μM) was added to these cultures for 3 days after which the Ara-C containing media was changed to fresh NB media. Experiments were done using cells at 7–10 DIV.

Unless indicated otherwise, the condition for heat shock was at 42°C for a specified time period beginning at 12–18 hr after the addition of riluzole. Cells were either harvested immediately for analysis of HSF1 or allowed to recover at 37° for 4 hr for analysis of Hsp70-firefly luciferase reporter gene expression and induction of the 72 kDa HSP 70 protein.

### Assay of Hsp70 promoter driven firefly luciferase reporter

We have constructed both the human and the mouse hsp 70 promoter driven-firefly luciferase reporter genes [23], [24], [55]. For construction of the human hsp 70-luciferase reporter (human hsp 70-luc), a 2.8 kb *BamH*1 restriction enzyme fragment of the human hsp 70 promoter from the pHBCAT construct [56] was ligated to the *Bgl*II linearized pGL3E vector (5064 bp) from Promega Inc. Proper orientation of the promoter was confirmed by restriction enzyme digestion and DNA sequencing. For construction of the mouse hsp 70 promoter-luciferase reporter (mouse hsp 70-luciferase), a 1,036 bp KpnI and NcoI restriction enzyme fragment from the construct pLHSEU4 [57] was ligated to the KpnI/NcoI digested pGL3E (5,006 bp). All constructs were confirmed by DNA sequencing. Quanlitatively similar results were obtained using the two reporter gene constructs in various human and rodent cell lines. For experiments described in this study, the mouse hsp 70 promoter-firefly luciferase reporter gene was used.

Cells were transfected with the Hsp70-firefly luciferase reporter DNA along with the internal control of phRLSV40 (synthetic humanized Renilla luciferase DNA) [23], [24]. Unless indicated otherwise, the amount of each DNA used was 0.5 μg/35 mm plate or 1.5 μg/60 mm plate, and the amount of Lipofectamine 2000 used (in μl) was 3× that of the total amount of DNA (in μg). 6 hr after DNA transfection, cells were trypsinized and plated into individual wells of a 96 Stripwell™ plate (Corning/Costar 9102); these identically transfected cells allowed for testing of the effects of riluzole and heat shock on reporter gene expression. To evaluate heat shock induction of the Hsp70-luciferase reporter gene, strips of 8 wells or designated wells of cells were placed in a 42°C incubator for 2 hr followed by recovery at 37°C for 4 hr prior to harvesting. The assay is robust and allowed for semi-high-throughput screening of the effects of drugs and treatment conditions on hsp70-reporter gene expression.

The Dual-Glo luciferase assay reagent system from Promega Inc. (E2920) was used to assay for first the firefly then the Renilla luciferase activity according to manufacturer's instructions. Luciferase activity was measured using the Perkin Elmer Victor 2 multiplate reader equipped with dual injectors. Result of the Hsp70-firefly luciferase activity was normalized against that of the Renilla luciferase, and to facilitate comparison across experiments for statistical analysis this ratio was set at 1 for the control. By normalizing the Hsp70-firefly luciferase activity against that of the Renilla luciferase internal control, we effectively negated experimental variables such as differences in transfection efficiency, cell number, as well as non-selective and toxic effects of the treatment conditions/reagents on gene expression. Statistical analysis was done by one-way ANOVA test using the GraphPad InStat program.

### Analysis of HSF1 and HSP70 by Western blotting

Whole cell and cytosol and nuclear extracts were prepared as previously described [22]. In some experiments (Fig. 5 and 6), cells were lysed using RIPA buffer in the presence of protease and phosphatase inhibitors. For Western blotting, aliquots of the cell extract containing the same amount protein (∼10 μg/lane) were loaded onto an 8% SDS-polyacrylamide gel, and proteins on the gel were transferred to a PVDF membrane. The membrane was probed with a 1∶10,000 dilution (overnight at 4°C) of a rabbit polyclonal antibody, RTG88, that we produced against a recombinant histidine-tagged human HSF1 protein. The antibody was diluted in Tris-buffered saline with 0.1% Tween 20 and 3% non-fat dry milk. This was followed by a 2 hr incubation at room temperature with a 1∶20,000 dilution of an affinity purified HRP-conjugated goat anti-rabbit IgG (Chemicon Int.), and detection by the Immobilon Western detection reagent (Millipore WBKLS05).

To assess the stoichiometry of HSF1, aliquots of cytosol and nuclear extracts containing 10 μg protein were incubated with 2 mM glutaraldehyde at room temperature for 10 min followed by quenching of the protein crosslinking reaction with the addition of 100 mM lysine [22]. Samples were subjected to SDS-PAGE (4–12% acrylamide gel) followed by immuno-Western blot probing for HSF1 as described above.

For immuno-Western blot detection of the HSP70 protein, membrane was incubated with a rabbit polyclonal antibody from Stressgen (SPA812, 1∶10,000 dilution) protein at 4°C overnight followed by horseradish peroxidase (HRP) conjugated secondary antibody for 2 hr at room temperature. The antibody was diluted in Tris-buffered saline with 0.1% Tween 20 and 3% non-fat dry milk, and the immunoblot was probed using the Millipore Immobilon Western blot reagent.

### Immunochemical staining for HSF1

Cells in 60 mm plates were fixed with 4% paraformaldehyde for 30 min at 4°C, permeabilized with 0.1% Triton×100 in phosphate-buffered saline (PBS) for 30 min at 4°C, and washed 3× with cold PBS. Wax pen encircled areas (∼1cm in diameter) of the fixed and permeabilized cells were overlaid with a 1∶1,000 dilution of the RTG88 anti-HSF1 antibody and incubated at 4°C for 1 hr. After washing off the primary antibody, cells were overlaid with a 1∶200 dilution of FITC-conjugated goat anti-rabbit IgG and incubated at 4°C for 1 hr. When indicated, nuclei were counter stained with 8 μM of Hoechst 33342. Cells were mounted in an anti-fade/glycerol solution and viewed using a Nikon Diaphot 300 microscope. Phase and fluorescent images were captured with a SPOT camera system (Diagnostic Instruments, Inc., Sterling Heights, MI).

### Cell viability assay of the cytoprotective activity of riluzole treatment and conditioning heat shock

Riluzole was added to individual wells to final concentrations as indicated and incubated at 37°C for 16 hr. For conditioning heat shock, designated strips of cells were heat shocked at 42°C for 2 hrs followed by recovery at 37°C. To test for cell survival under conditions of oxidative stress, 20 μM sodium arsenite was added to designated wells of cells at 24 hr after conditioning heat shock and incubated at 37°C for 24 hr. Viability of the cells were determined usin*g the* CellTiter-Glo (G7571) luminescent reagent from Promega Inc. Cell viability, relative to that of the untreated control, is plotted as a function of the concentration of riluzole added.

### Assessment of the turnover of HSF1 by [^35^S]methionine labeling and chase

Confluent cultures in 35 mm plates were refurbished with serum-free medium. Cells were pulse labeled with 300 μCi/ml of [^35^S]methionine/cysteine (Amersham Pro-Mix, a 70∶30% mixture of [^35^S]methionine and [^35^S]cysteine) for 2 hr at 37°C. At the end of this labeling periods, cells were rinsed extensively and refurbished with DMEM containing 2 mM each of cysteine and methionine to initiate the chase. To test for the effects of riluzole on the turnover of [^35^S]HSF1, it was added to designated plates to a final concentration of 2 μM at the beginning of the chase. Cells were harvested at 0, 4, 8, 12 and 24 hr after initiation of the chase in the absence versus in the presence of 2 μM riluzole. The cell pellets were lysed in 200 μl of RIPA buffer, and an aliquot (∼50 μl) of the RIPA extract was used for the immunoprecipitation of HSF1. For this, 2 μl of the RTG88 anti-HSF1 antibody was added to each sample and incubated at 4°C overnight. The antigen-antibody complex was immunoprecipitated by the addition of insoluble protein A and incubation at room temperature for 2 hr. The immunoprecipitate was collected by centrifugation and washed 3× each with 200 μl of RIPA buffer, and the amount of radioactivity determined by liquid scintillation counting.

### RT-PCR quantitation of mRNAhsf1

RNA was isolated from cells grown in 60 mm plates using the TRIzol reagent from Invitrogen Inc. RT-PCR was done using the 2-step procedure (cDNA synthesis with M-MLV reverse transcriptase, followed by PCR amplification with HSF1-specific primers). The forward and reverse primers of HSF1 were: CATGAGAATGAGGCTCTGTG and CTACGCTGAGGCACTTTTCA. The PCR reaction was for 30 cycles using Platinum PCR SuperMix from Invitrogen Inc. The amplied HSF1 product is 642bp.
